# Activation of the reverse transsulfuration pathway through NRF2/CBS confers erastin-induced ferroptosis resistance

**DOI:** 10.1038/s41416-019-0660-x

**Published:** 2019-12-10

**Authors:** Nan Liu, Xiaoli Lin, Chengying Huang

**Affiliations:** Division of Obstetrics and Gynecology, Nanfang Hospital, Southern Medical University, Guangzhou, 510515 China

**Keywords:** Ovarian cancer, Cell death

## Abstract

**Background:**

Ferroptosis is an iron-dependent, lipid peroxide-mediated cell death that may be exploited to selective elimination of damaged and malignant cells. Recent studies have identified that small-molecule erastin specifically inhibits transmembrane cystine–glutamate antiporter system x_c_^−^, prevents extracellular cystine import and ultimately causes ferroptosis in certain cancer cells. In this study, we aimed to investigate the molecular mechanism underlying erastin-induced ferroptosis resistance in ovarian cancer cells.

**Methods:**

We treated ovarian cancer cells with erastin and examined cell viability, cellular ROS and metabolites of the transsulfuration pathway. We also depleted cystathionine β-synthase (CBS) and NRF2 to investigate the CBS and NRF2 dependency in erastin-resistant cells.

**Results:**

We found that prolonged erastin treatment induced ferroptosis resistance. Upon exposure to erastin, cells gradually adapted to cystine deprivation via sustained activation of the reverse transsulfuration pathway, allowing the cells to bypass erastin insult. CBS, the biosynthetic enzyme for cysteine, was constantly upregulated and was critical for the resistance. Knockdown of CBS by RNAi in erastin-resistant cells caused ferroptotic cell death, while CBS overexpression conferred ferroptosis resistance. We determined that the antioxidant transcriptional factor, NRF2 was constitutively activated in erastin-resistant cells and NRF2 transcriptionally upregulated CBS. Genetically repression of NRF2 enhanced ferroptosis susceptibility.

**Conclusions:**

Based on these results, we concluded that constitutive activation of NRF2/CBS signalling confers erastin-induced ferroptosis resistance. This study demonstrates a new mechanism underlying ferroptosis resistance, and has implications for the therapeutic response to erastin-induced ferroptosis.

## Background

System x_c_^−^ is a transmembrane cystine–glutamate antiporter that specifically imports extracellular l-cystine into cells in exchange for glutamate.^1,2^ Cystine is an oxidised, disulfide form of cysteine. Due to intracellular reducing context, cystine imported into the cell by system x_c_^−^ is reduced to cysteine, which is required for the synthesis of glutathione (GSH), the key cellular antioxidant component, maintaining intracellular redox homoeostasis.^3,4^ Many cells rely on system x_c_^−^ to take up cystine, but this uptake is the rate-limiting step in obtaining cysteine. Blocking or inhibiting this process causes cystine deprivation, and perturbs cellular redox state.^5^

System x_c_^−^ is a heterodimer composed of two distinct proteins: the substrate-specific subunit xCT also known as SLC7A11 (solute carrier family 7), which is responsible for the transporter function of system x_c_^−^, and heavy chain of 4F2 cell surface antigen (4F2hc) SLC3A2 (solute carrier family 3 member 2).^6,7^ Recent studies have identified that small-molecule erastin specifically inhibits xCT, resulting in depletion of cellular GSH, and iron-dependent cell death, designated as ferroptosis.^8^ Ferroptosis is featured by irresolvable lipid peroxidation, which is mechanistically triggered by ferrous iron (Fe^2+^)-mediated Fenton reaction.^9–12^ Deprivation of cysteine by system x_c_^−^ blockage leads to ferroptosis through GSH depletion and lipid peroxidation in some cell contexts, while some types of cells are tolerant to system x_c_^−^ inhibition.

In addition to importing cystine by system x_c_^−^, mammalian cells can obtain cysteine through the reverse transsulfuration pathway, which is the only route for cysteine biosynthesis.^13–15^ The pathway channels dietary-derived methionine to S-adenosyl homocysteine and homocysteine. Cystathionine β-synthase (CBS) catalyses the condensation of homocysteine with serine to generate cystathionine, which cystathione γ-lyase (CSE) converts to cysteine.^16^ Disruption of the transsulfuration pathway is associated with pathological conditions of several diseases such as vascular dysfunction^17^ and Huntington’s disease.^18,19^ Previous studies have reported that certain types of cancer cells, which rely on system x_c_^−^ for cystine uptake due to their inability to synthesise cysteine via the transsulfuration pathway, are particularly sensitive to erastin insult.^20–22^ Therefore, understanding the molecular mechanisms underlying the crosstalk between ferroptosis and the transsulfuration pathway will contribute to the design of therapeutic approaches to regulate cancer cell fate.

In this study, we observed erastin-induced ferroptosis resistance in ovarian cancer cells. We found that the transsulfuration pathway was elevated due to CBS upregulation. Enhanced flux through the transsulfuration pathway provided cells with cysteine, which compensated the shortage of cysteine by system x_c_^−^ blockage. We demonstrated that constitutive activation of NRF2/CBS signalling conferred erastin-induced ferroptosis resistance.

## Methods

### Cell culture and reagents

Human ovarian cancer SKOV3 and OVCA429 cells were maintained in RPMI 1640 (SKOV3 cells) or DMEM (OVCA429 cells) medium supplemented with 10% foetal bovine serum and 1% penicillin/streptomycin at 37 °C with 5% CO_2_ in a humidified atmosphere. To establish erastin-resistant cells, SKOV3 cells were treated with 10 µM erastin for 7 days, OVCA429 cells were treated with 20 µM erastin for 5 days and the remaining cells were washed and cultured in fresh medium. Cell viability was analysed by using the Cell Counting Kit-8 (CCK-8) (Sigma #96992) according to the manufacturer’s instructions. Liproxstatin-1 (Lipo-1), 7-Cl-O-Nec1 (Nec-1s) and Z-VAD-FMK were obtained from Selleckchem; erastin, ferrostatin-1 (Fer-1), N-acetyl cysteine (NAC), desferrioxamine (DFO), Trolox, necrosulfonamide (NSA), sulfasalazine (SAS) RSL3 and FIN56 were purchased from Sigma. CM-H_2_DCFDA and C11-BODIPY (581/591) were purchased from Molecular Probes, Invitrogen.

### ROS analysis

Cells were incubated with 5 µM CM-H_2_DCFDA for ROS, or 5 µM C11-BODIPY (581/591) for lipid ROS in the dark for 30 min at 37 °C. Cells were then harvested and resuspended in phenol-red-free medium. Fluorescence was analysed on BD FACsCalibur system with CellQuest Pro software.

### Lipid peroxidation assay

Malondialdehyde (MDA), the end product of lipid peroxidation, was assessed by using a lipid peroxidation (MDA) assay kit (#MAK085, Sigma). Cells were homogenised on ice in MDA lysis buffer, followed by centrifugation at 13,000 × *g* for 3 min. MDA in cell lysate reacts with thiobarbituric acid (TBA) solution to generate MDA-TBA adduct, which is colorimetric (OD = 532 nm) and is proportional to the MDA present.

### Glutathione (GSH) assay

Cells were collected and homogenised in a 5% 5-sulfosalicylic acid (SSA) solution. The reduced GSH concentration in cell lysates was measured by using the Reduced Glutathione (GSH) Assay kit (# K464-100, BioVision) as per the manufacturer’s instructions. The results were normalised to total protein concentration for each sample.

### Glutamate release assay

The release of glutamate from cells into the extracellular medium was assayed as described.^23^ Cells were washed with cystine uptake buffer (137 mM choline chloride, 3 mM KCl, 1 mM CaCl_2_, 1 mM MgCl_2_, 5 mM d-glucose, 0.7 mM K_2_HPO_4_, 10 mM HEPES and 300 µM cystine, pH 7.4), then incubated with uptake buffer containing DMSO or 10 µM erastin for 60 min at 37 °C. Glutamate in cell medium was detected by using Amplex Red Glutamic Acid/Glutamate Oxidase Assay kit (# A-12221, Thermo Fisher) as per the manufacturer’s instructions. Cell number was quantified by trypan blue exclusion assay. Glutamate release was normalised to cell number.

### Cystine uptake assay

Cells were washed three times with pre-warmed sodium-free uptake buffer (137 mM choline chloride, 3 mM KCl, 1 mM CaCl_2_, 1 mM MgCl_2_, 5 mM d-glucose, 0.7 mM K_2_HPO_4_ and 10 mM HEPES, pH 7.4), then incubated with 0.5 ml of pre-warmed uptake buffer at 37 °C for 10 min. The buffer was then replaced with 0.5 ml of [^14^C]cystine (0.2 μCi/mL) uptake buffer and incubated at 37 °C for 3 min. Cells were rapidly washed three times with ice-cold uptake buffer and lysed in 0.5 ml of 0.1 M NaOH. The radioactive counts per minute were assessed by a scintillation counter.^24^

### Metabolomic profiling

Cell pellets (≥2 × 10^6^ cells) were flash frozen in liquid nitrogen and stored at −80 °C until processing. Metabolites were profiled by Dian Diagnostics Co. Ltd. (Zhejiang, China). Samples were prepared by using aqueous methanol extraction process to remove the protein fraction while allowing maximum recovery of small molecules. For quality control (QC) purpose, a recovery standard was included before the extraction process. The extract was divided into four fractions: one for analysis by UPLC/MS/MS with positive ion mode electrospray ionisation (ESI) (positive mode), one for UPLC/MS/MS with negative ion mode ESI (negative mode), one for GC/MS and one for backup. Each sample was processed to remove the organic solvent, and prepared for either UPLC/MS/MS or GC/MS. For UPLC/MS/MS, the sample extracts were reconstituted in acidic or basic LC-compatible solvents. The acidic extracts were analysed by using positive mode, and gradient eluted by using water and methanol containing 0.1% formic acid, while the basic extracts were analysed by using negative mode and eluted by using water/methanol containing 6.5 mM ammonium bicarbonate. The MS analysis alternated between MS and data-dependent MS^2^ scans using dynamic exclusion. For GC/MS analysis, samples were derived using bistrimethyl-silyl-triflouroacetamide (BSTFA), and analysed on a Thermo-Finnigan Trace DSQ fast-scanning single-quadrupole mass spectrometer using electron impact ionisation. Raw data were extracted and peak-identified using Metabolon’s hardware and software. Compounds were identified by comparison with library entries of purified standards on both the LC and GC platforms. Each biochemical value was normalised to cell number and scaled to its median value. The unpaired *t* test for differential abundance with a standard two-tailed and two-sample *t* test was performed using mattes function in MATLAB on each metabolite to calculate the Wilcoxon *t* statistic for two groups being compared. The resultant *p*-value of less than 0.05 was selected as significant. The experiment was conducted in biological quintuplicate.

### Western blotting

Cell pellets were lysed in RIPA buffer (25 mM Tris-HCl, pH = 7.5, 150 mM NaCl, 0.1% Nonidet P-40, 0.5% sodium deoxycholate and 0.1% SDS) supplemented with protease inhibitor cocktail (Sigma) and phosphatase Inhibitor Cocktail 2 and 3 (Sigma). The lysates were cleared and subjected to SDS-PAGE and transferred onto nitrocellulose membranes. The following antibodies were used: anti-CBS (#14782, Cell Signaling Technology [CST]), anti-CSE (#30068, CST), anti-NRF2 (#12721, CST), and anti-GAPDH (sc-32233, Santa Cruz Biotechnology).

### siRNA reverse transfection and lentivirus infection

About 10 nM of siRNA in OptiMEM (250 µl) was mixed with 250 µl of OptiMEM with 2.5 µl of Lipofectamine RNAiMAX (Invitrogen) for 20 min, then with 1.5 ml of cell suspension in regular medium. A total of 200,000 cells per well were seeded to six-well plates. Cells were then analysed at indicated time points. All siRNAs were purchased from Sigma: siCtrl (SIC001), siCBS (SASI_Hs01_00214623) and siNRF2 (SASI_Hs01_00182393). Lentiviruses carrying CBS or empty vector were previously described.^25^ HEK293T cells were transfected by pCDH-hCBS or pCDH vector constructs along with packaging constructs as per Lipofectamine 2000 manual.

### qRT-PCR

RNA was extracted using Trizol reagent (Invitrogen). cDNA was synthesised using SuperScript™ III reverse transcriptase and oligo-dT (Life Technologies). qPCR was performed using the iTaq universal SYBR green supermix (Bio-rad). GAPDH was used for normalisation in all qPCR assays. Fold changes were analysed by the 2–ΔΔCT method for relative quantification. The primers used for qPCR were as follows: CBS: 5′-GGCCAAGTGTGAGTTCTTCAA-3′ and 5′-GGCTCGATAATCGTGTCCCC-3′; CSE: 5′-CATGAGTTGGTGAAGCGTCAG-3′ and 5′-AGCTCTCGGCCAGAGTAAATA-3′; GAPDH: 5′-CATGGGTGTGAACCATGAGA-3′ and 5′-CAGTGATGGCATGGACTG-TG-3′.

### Measurement of hydrogen sulfide (H_2_S)

H_2_S was determined in erastin-resistant cells following the protocol as previously described.^26,27^ Cells were lysed in a lysis buffer (potassium phosphate buffer 100 mM, pH 7.4, sodium orthovanadate 10 mM and proteases inhibitors). Protein concentration was determined by Pierce BCA Protein Assay Kit. Cell lysates were incubated with a reaction mixture containing pyridoxal-5-phosphate (2 mM) at 37 °C for 30 min. Next, trichloroacetic acid solution (10%) was added to each sample followed by zinc acetate (1%). Then, N,N-dimethyl-p-phenylendiamine sulfate (DPD, 20 mM) in HCl (7.2 M) and FeCl_3_ (30 mM) in HCl (1.2 M) were added, and the absorbance of the solutions was measured at a wavelength of 650 nm. Aminooxyacetic acid (AOAA, 1 mM), a CBS inhibitor, was added to the reaction mixture in some samples, which served as a positive control of CBS inhibition. H_2_S production was plotted against a calibration curve of NaHS (3–250 μM). Data were presented as nanomoles per milligram of total protein per minute.

### Plasmid constructions and reporter activity assay

The 1200-bp DNA fragment containing the CBS promoter region from −1000 to +200 bp was amplified from SKOV3 Era-R cell genomic DNA by PCR with the following primers: CBS-*Kpn*I forward, 5′-GGTACCGACATTTAATTCTAATTCACGTCTC-3′ and CBS-*Bgl*II reverse, 5′-AGATCTGTCCAGAGAGGGGAGCGAGTCTCGG-3′. This fragment was cloned into the pGL3-basic luciferase reporter vector (Promega), using *Kpn*I and *Bgl*II restriction sites. This construct containing the human CBS promoter was named pGL3-phCBS. The NRF2-binding antioxidant response element (ARE) deletion mutant of CBS promoter–luciferase construct pGL3-phCBS-ΔARE was generated by the overlapping PCR technique and ligated into pGL3-basic luciferase reporter vector (Promega). The following two primer sets were used: (I) CBS-*Kpn*I forward primer and reverse primer, 5′-CGGCGACCCCGGGGTGGGGACCCACGGCGA-3′ and (II) forward primer, 5′-TCGCCGTGGGTCCCCACCCCGGGGTCGCCG-3′ and CBS-*Bgl*II reverse primer. The pCDNA3-Myc3-NRF2 was a gift from Yue Xiong (Addgene plasmid #21555). The pCDNA3 empty vector was made from pCDNA3-Myc3-NRF2 by removing Myc3-NRF2 sequence and religating the vector. A pCH110 plasmid encoding β-galactosidase (β-gal) was purchased from Amersham. To determine the effect of NRF2 on the CBS promoter activity, SKOV3 Era-R cells were seeded in 24-well plates and transiently cotransfected with each reporter plasmid, pCDNA3-Myc3-NRF2 or pCDNA3 empty vector, and pCH110 plasmid using Lipofectamine 2000 reagent (Invitrogen). After incubation for 24 h, transfected cells were subjected to a luciferase activity assay using Luciferase Assay System (Promega). Relative luciferase activity was normalised to β-gal activity as described previously.

### Statistical analysis

All of the results are mean values ± SD of three biological replicates. One-way ANOVA followed by Dunnett’s multiple comparisons test was performed using GraphPad Prism version 8.0.0 for Windows (GraphPad Software, San Diego, California USA, www.graphpad.com).

## Results

### Lethal dose of erastin induces ferroptosis resistance in ovarian cancer cells

**Fig. 1:**
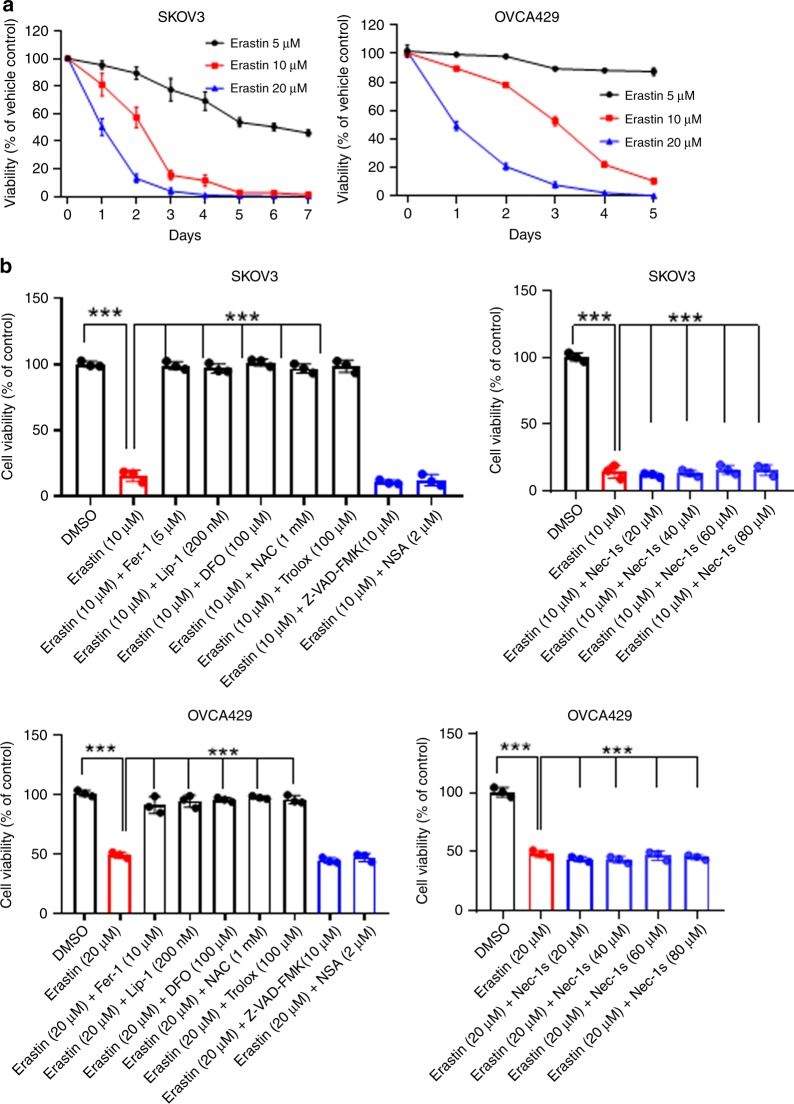

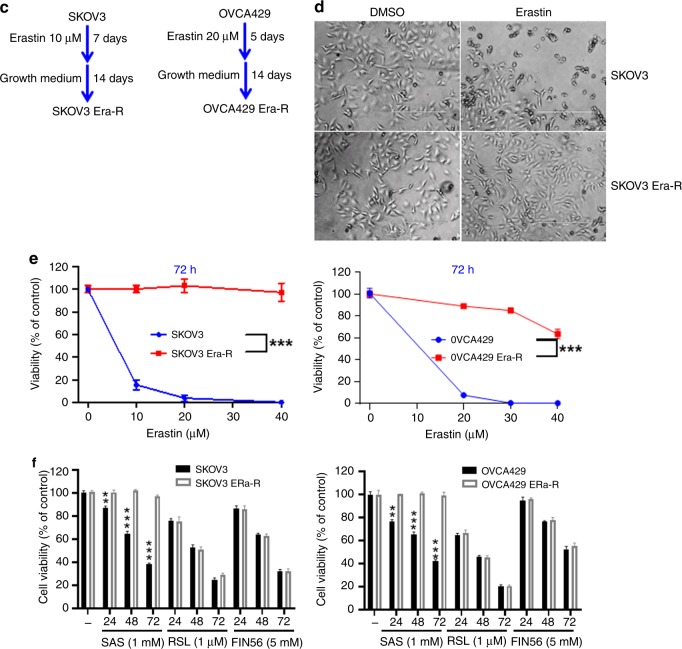
Lethal dose of erastin induces ferroptosis resistance in ovarian cancer cells. **a** SKOV3, OVCA429 cells were sensitive to erastin-induced death. SKOV3, OVCA429 cells were treated with erastin (0, 5, 10 and 20 μM) for the indicated time points, and cell viability was analysed. **b** Fer-1, Lip-1, DFO, NAC and Trolox rescued erastin-induced death, confirming erastin-induced ferroptosis in SKOV3 and OVCA429 cells. Cells were treated with erastin in the absence or presence of indicated inhibitors (Fer-1, Lipo-1, BSO, NAC, Troxl, z-VAD-FMK, NAS and Nec-1s) for 24 h, and cell viability was assayed, ***p < 0.0001. **c** Outline of the establishment of SKOV3 and OVCA429 erastin-resistant cells. **d** SKOV3 Era-R cells were resistant to lethal dosage of erastin. SKOV3 and SKOV3 Era-R cells were treated with erastin 20 μM for 24 h. Cell viability was monitored by light microscopy (scale bar = 400 μm, magnification: ×10). **e** SKOV3 Era-R, OVCA429 Era-R cells were resistant to erastin insult. Cells were treated with erastin (0, 10, 20 and 40 μM) for 72 h, and cell viability was examined, ***p < 0.0001. **f** SKOV3, SKOV3 Era-R, OVCA429 and OVCA429 Era-R cells were either untreated or treated with SAS (1 mM), RSL (1 μM) or FIN56 (5 μM) for the indicated time, cell viability was examined. **p < 0.001, ***p < 0.0001 as compared with untreated control.

We initially set out to investigate the ferroptosis susceptibility of ovarian cancer cells in response to erastin. SKOV3 and OVCA429 cells were sensitive to erastin-induced cell death (Fig. 1a). The ferroptosis inhibitors ferrostatin-1 (Fer-1), liproxstatin-1 (Lip-1), iron chelator deferoxamine (DFO) and the antioxidants NAC and Trolox^8,28^ rescued the lethal erastin, while the apoptosis inhibitor Z-VAD-FMK,^29^ and the necroptosis inhibitors necrosulfonamide (NSA), 7-Cl-O-Nec1 (Nec-1s)^28^ failed to block erastin-induced death (Fig. 1b), indicating that erastin is a potent ferroptosis inducer in the tested cell lines. We noticed that some cells still survived even when treated with lethal dose of erastin for 5–7 days. These cells were recovered in fresh growth medium and displayed significant resistance to the erastin insult, therefore, they were named as SKOV3 Era-R and OVCA429 Era-R for erastin-resistant cells (Fig. 1c–e). The established erastin-resistant cell lines also displayed resistance to sulfasalazine (SAS), which like erastin, triggers ferroptosis through inhibition of system X_c_^−8^ (Fig. 1f). We tested the sensitivity of these erastin-resistant cell lines to the other types of ferroptosis inducers, RSL3 and FIN56,^30^ which induce ferroptosis by inhibiting the glutathione peroxidase GPX4, a downstream antioxidant enzyme of system X_c_^−^. Over 72 h, RSL3- and FIN56-induced growth inhibition in SKOV3 Era-R and OVCA429 Era-R cells was on par with that in the parental SKOV3 and OVCA429 cells, respectively (Fig. 1f). This observation indicated that resistance was likely via modulation of upstream GPX4.

### Adaption to erastin insult reprograms cellular cysteine metabolism

**Fig. 2:**
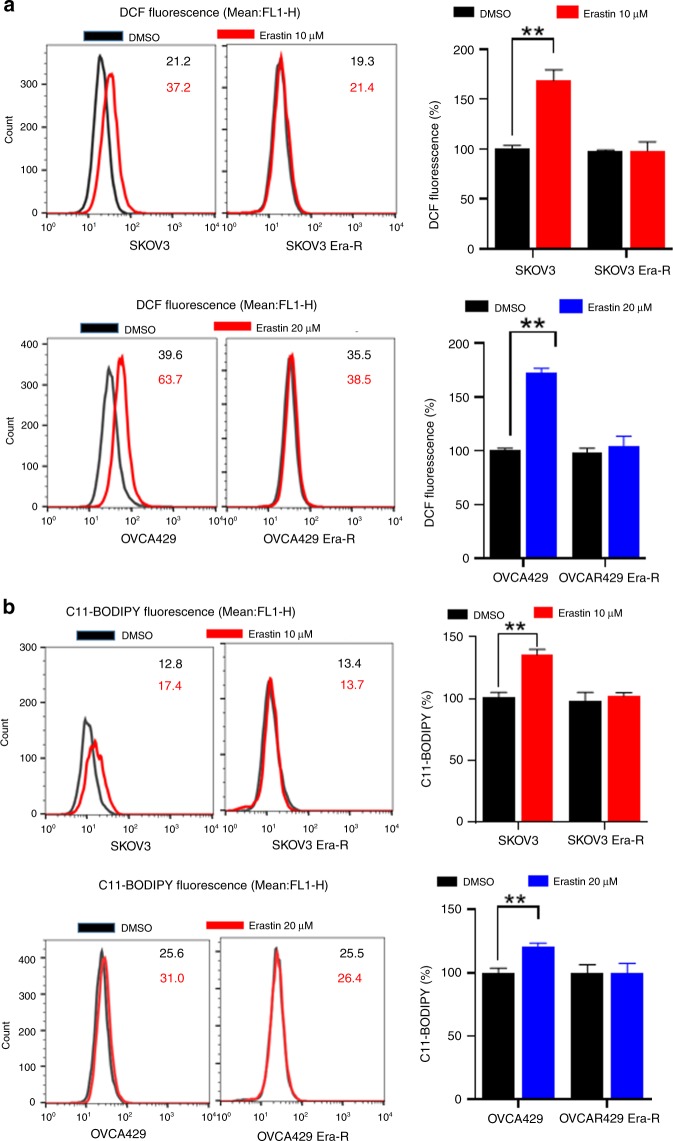

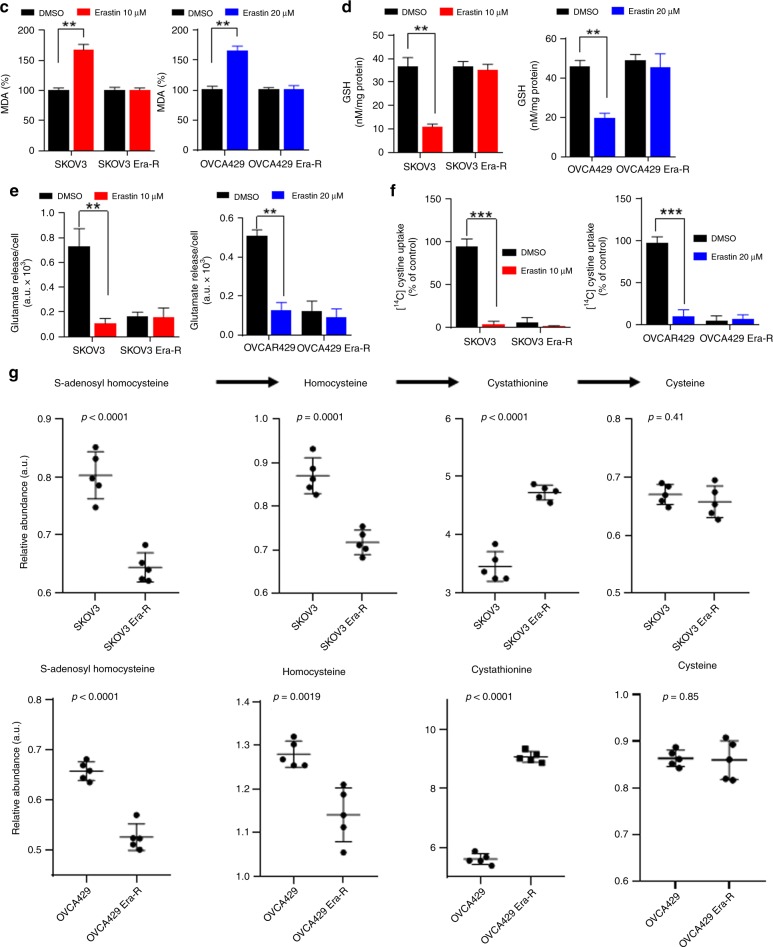
Adaption to erastin insult reprograms cellular cysteine metabolism. **a–d** Cells were treated with erastin or DMSO for 8 h. Cellular and lipid ROS were assayed by flow cytometry using H_2_DCFDA (**a**) and C11-BODIPY (**b**), respectively. The lipid peroxidation marker, MDA (**c**), and GSH content (**d**) were measured, **p < 0.001. **e** Cells were treated with DMSO or erastin for 8 h. Glutamate release from cells to culture medium was examined, **p < 0.001. **f** Cells were exposed to erastin for 8 h. The activity of cystine uptake was measured, ***p < 0.001. **g** Analysis of metabolites of the transsulfuration pathway. Intracellular S-adenosyl homocysteine, homocysteine, cystathionine and cysteine are shown.

Erastin inhibits the transmembrane cystine–glutamate antiporter system X_c_^−^, blocks cystine influx, leading to intracellular cysteine shortage and consequently depletion of GSH, causing extensive lipid peroxidation, a featured event in ferroptosis.^8^ To gain insight into the erastin resistance, we treated cells with erastin and analysed cellular ROS and lipid peroxidation by flow cytometry using the fluorescent probes H_2_DCFDA and C11-BODIPY, respectively. As expected, both SKOV3 and OVCA429 cells showed an increase in fluorescence after 8 h of treatment with erastin, whereas no increase in fluorescence was observed in SKOV3 Era-R and OVCA429 Era-R cells (Fig. 2a, b). We further examined the accumulation of the end product of lipid oxidation, malondialdehyde (MDA).^31^ As compared with vehicle-treated cells, erastin treatment caused much increase in MDA in SKOV3 and OVCA429 cells, but not in SKOV3 Era-R and OVCA429 Era-R cells (Fig. 2c). We also quantified GSH content in cells upon erastin treatment. Erastin-induced downregulation of GSH was clearly observed in SKOV3 and OVCA429 cells, while GSH content in SKOV3 Era-R and OVCA429 Era-R cells remained unaltered (Fig. 2d). These data together suggested that erastin-resistant cells adapted to erastin insult through a mechanism to rebuild up GSH homoeostasis. We monitored glutamate release to culture medium and confirmed that erastin acts via system X_c_^−^ inhibition (Fig. 2e); however, both SKOV3 Era-R and OVCA429 Era-R cells exhibited minimal glutamate release even without erastin treatment (Fig. 2e). We further compared the activity of system X_c_^−^ in parental cells and erastin-resistant cells by determining the uptake of [^14^C] cystine into cells. Erastin treatment markedly blocked the cystine uptake in parental cells, whereas cystine uptake was severely blocked in erastin-resistant cells even without erastin treatment (Fig. 2f). These data supported the previous observation that erastin irreversibly inhibits system X_c_^− 32^ and indicated the impaired system X_c_^−^ in erastin-resistant cells. Since system X_c_^−^ functions as a primary source of cysteine for GSH biosynthesis, these erastin-resistant cells might have developed an alternative source to provide cysteine, compensating the shortage of cystine influx resulting from system X_c_^−^ blockage. We wondered whether erastin-resistant cells rely on the activation of transsulfuration pathway to synthesise cysteine. To this end, we performed global metabolic profiling on cellular extracts from SKOV3, SKOV3 Era-R, OVCA429 and OVCA429 Era-R cells and analysed metabolites in the transsulfuration pathway. There was no change in the level of cysteine in both parental and erastin-resistant cells. We observed an elevated flux through the transsulfuration pathway upstream of cysteine synthesis in erastin-resistant cells. The levels of S-adenosyl homocysteine and homocysteine were decreased in both SKOV3 Era-R and OVCA429 Era-R cells as compared with their parental cells, but a significant increase in cystathionine levels was detected in erastin-resistant cells (Fig. 2g). These data suggested that the upregulated transsulfuration pathway for cysteine synthesis in erastin-resistant cells compensated for cystine deprivation by system X_c_^−^ blockage.

### Constitutive upregulation of CBS confers ferroptosis resistance

**Fig. 3:**
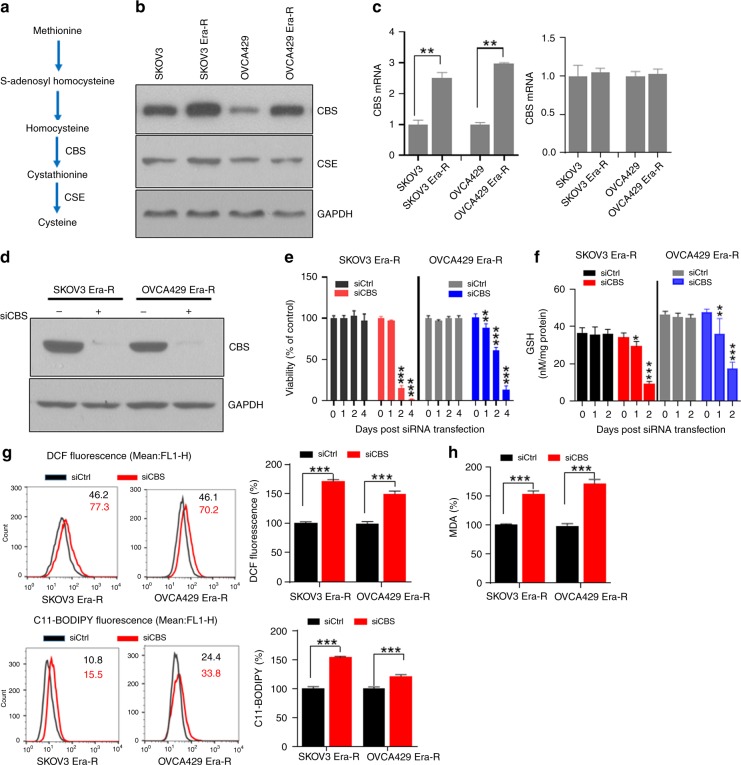

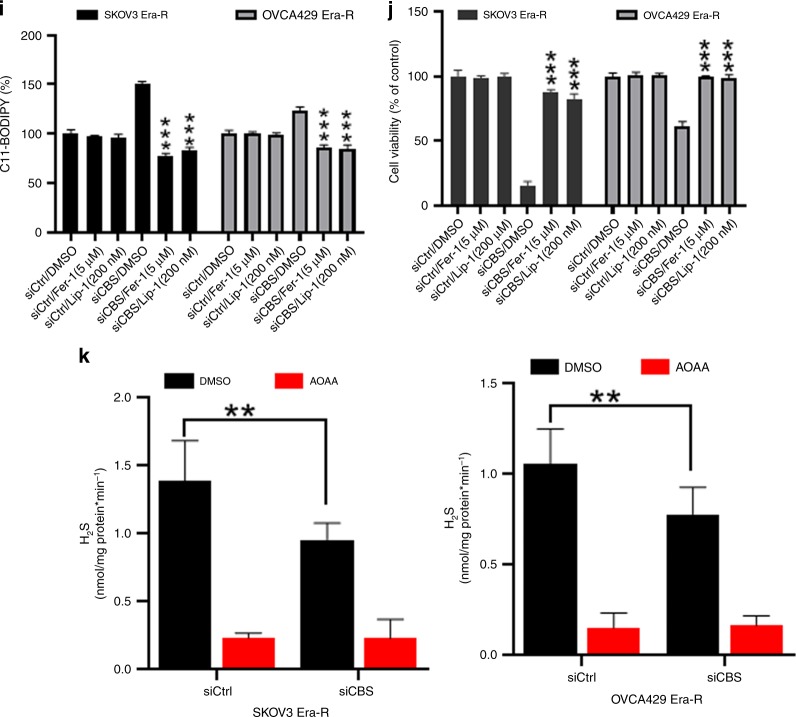
Constitutive upregulation of CBS confers ferroptosis resistance. **a** An overview of the transsulfuration pathway channels methionine towards cysteine. **b** Cells were lysed and analysed for CBS and CSE expression by western blotting. **c** Comparison of CBS and CSE mRNA levels in erastin-resistant cells and their parental cells, **p < 0.001. **d** Cells were transfected with either scramble control siRNA or CBS siRNA for 48 h. CBS knockdown was validated by western blotting. **e** Cells were transfected with either scramble control siRNA or CBS siRNA. Cell viability was examined over 4 days, **p < 0.001, ***p < 0.0001 as compared with the group at day 0 of transfection. **f** Cells were transfected with indicated siRNAs; GSH content was measured over 48 h, *p < 0.01, **p < 0.001, ***p < 0.0001 as compared with the group at day 0 of transfection. **g**, **h** Cells were transfected with indicated siRNAs for 48 h; cellular ROS, lipid ROS (**g**) and MDA (**h**) were measured, ***p < 0.0001. **i**, **j** Cells were transfected with siRNAs for 24 h, followed by DMSO, Fer-1 or Lip-1 treatment for 24 h. Lipid ROS (**i**) and cell viability (**j**) were measured, ***p < 0.0001. **k** Basal level of hydrogen sulfide (H_2_S) in cell homogenates was measured. The addition of CBS inhibitor (AOAA, 1 mM) served as a positive control for CBS inhibition. Data were presented as nanomoles per milligram of total protein per minute, **p < 0.001 as compared with the group with siCtrl and DMSO treatment.

Regulation of metabolic flux through the transsulfuration pathway is well controlled at enzymatic reaction level. Two main enzymes, CBS, which catalyses the condensation of homocysteine to generate cystathionine, and CSE, which metabolises cystathionine into cysteine, are subject to regulatory control (Fig. 3a). To understand the mechanism underlying the upregulated transsulfuration pathway, we compared the expressions of CBS and CSE in parental cells and erastin-resistant cells. High levels of both protein and mRNA of CBS were found in erastin-resistant cells, while CSE protein did not show detectable difference between parental and erastin-resistant cells (Fig. 3b, c). Since the function of system X_c_^−^ is already blocked in erastin-resistant cells, as indicated by the inhibition of glutamate release to cell culture and the blockage of cystine uptake in Fig. 2e, f, we reasoned that CBS deficiency may cause cell death. Indeed, we observed that knocking down CBS by siRNA in erastin-resistant cells (Fig. 3d) triggered cell death (Fig. 3e). To clarify whether the CBS RNAi-induced cell death was mechanistically through ferroptosis pathway, we monitored the change in cellular GSH content, redox status and lipid peroxidation when CBS was depleted. As shown in Fig. 3f, GSH depletion was observed as early as 24 h after siRNA transfection, and the decrease of GSH was reversely correlated with the increase in cell death. Cellular ROS, as indicated by DCF fluorescence, and lipid peroxidation, as indicated by C11-BODIPY fluorescence (Fig. 3g) and MDA (Fig. 3h) production, were markedly enhanced by CBS knockdown. Furthermore, cellular lipid peroxidation was attenuated by the addition of ferroptosis inhibitors, Fer-1, as well as Lip-1 (Fig. 3i). CBS RNAi-induced cell death was also blocked by Fer-1 and Lip-1 (Fig. 3j), indicating that inhibition of CBS in the context of system X_c_^−^ blockage caused ferroptosis. CBS is a primary enzyme to catalyze the production of H_2_S,^33,34^ which has been reported to contribute to cellular GSH content and scavenge lipid peroxidation.^35,36^ To assess whether CBS depletion by RNAi was accompanied with alteration of H_2_S production, we quantified H_2_S levels in erastin-resistant cells receiving siCtrl or siCBS RNAs. Treatment of siCtrl cells with a CBS inhibitor, AOAA dramatically reduced the basal level of H_2_S, confirming the essential role of CBS in H_2_S production. Basal H_2_S was decreased ~1.5–1.6-fold in CBS-knockdown cells, when compared with siCtrl cells (Fig. 3k). These data together suggested that erastin-induced ferroptosis resistance was dependent on the upregulation of CBS.

### CBS overexpression elevates the flux through the transsulfuration pathway and mitigates ferroptosis

**Fig. 4:**
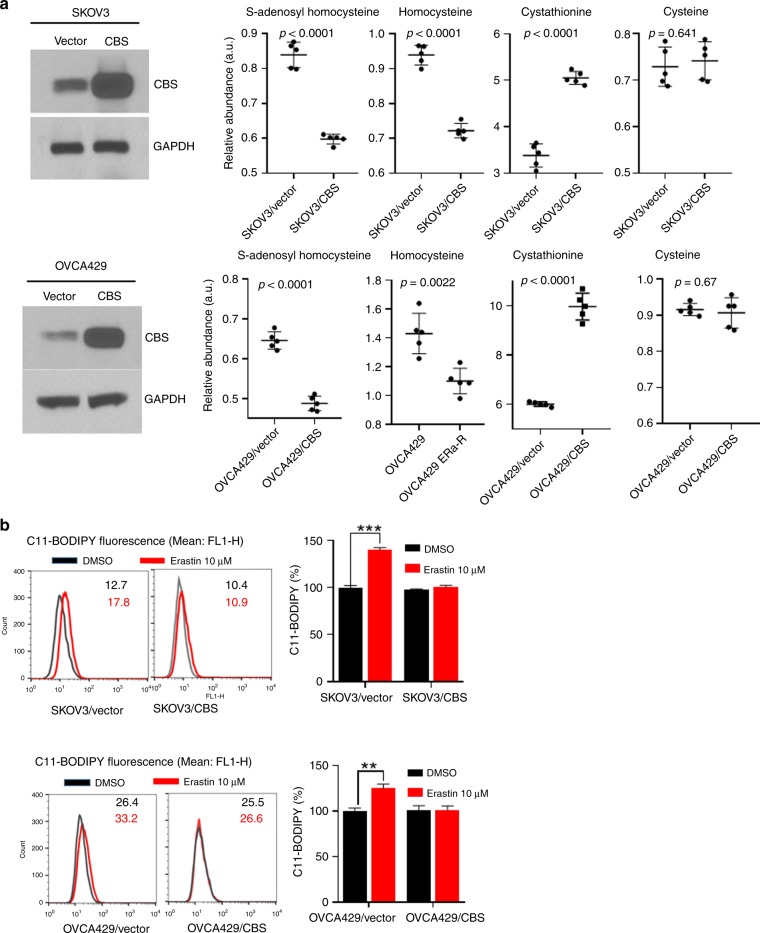

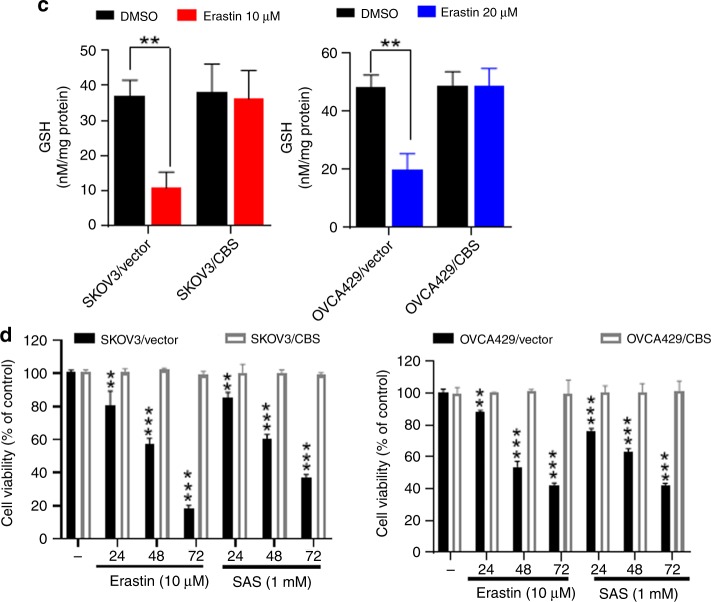
CBS overexpression elevates the flux through the transsulfuration pathway and mitigates ferroptosis. **a** Cells were infected with lentivector or lenti-CBS for 48 h. Left panel: CBS expression was analysed by western blotting. Right panel: cellular S-adenosyl homocysteine, homocysteine, cystathionine and cysteine were assessed. **b**, **c** Cells were infected with lentivector or lenti-CBS for 48 h, lipid ROS was analysed using C11-BODIPY probe (**b**), cellular GSH content was measured (**c**), **p < 0.001, ***p < 0.0001. **d** Cells were infected with lentivector or lenti-CBS for 48 h, followed by erastin (10 μM) or SAS (1 mM) treatment over 72 h. Cell viability was examined, **p < 0.001, ***p < 0.0001.

When CBS catalyses the initial and rate-limiting step of the transsulfuration pathway,^37^ we hypothesised that overexpression of CBS might increase the flux through the transsulfuration pathway, provide cells with alternative source of cysteine, thus rendering tolerance to cystine deprivation, as caused by erastin insult. To test this hypothesis, we overexpressed CBS in parental SKOV3 and OVCA429 cells (Fig. 4A). Metabolite profiles revealed the decrease in S-adenosyl homocysteine and homocysteine levels and the increase of cystathionine in CBS-overexpressed cells (Fig. 4a), indicating the enhanced flux of metabolites in the transsulfuration pathway. As expected, CBS overexpression dampened erastin-induced lipid peroxidation (Fig. 4b). GSH content was not much disturbed in CBS-overexpressing cells by erastin treatment (Fig. 4c). Strikingly, ferroptotic cell death induced by system X_c_^−^ inhibitor erastin, as well as SAS, was also rescued by CBS overexpression (Fig. 4d). Taken together, these data suggested that elevation of CBS mitigated ferroptosis by cystine deprivation via the enhanced transsulfuration pathway.

### Activated NRF2 upregulates CBS

**Fig. 5:**
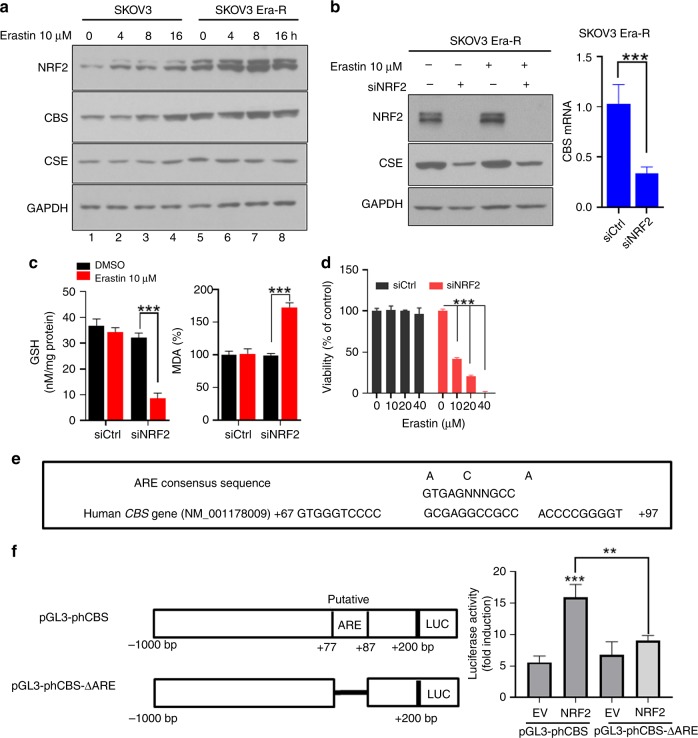
Activated NRF2 upregulates CBS. **a** SKOV3 and SKOV3 Era-R cells were treated with erastin (10 μM) for the indicated time; protein abundance was examined by western blotting. **b** SKOV3 Era-R cells were transfected with siCtrl or siNRF2 for 48 h, then treated with or without erastin (10 μM) for 24 h. Left panel: protein analysis by western blotting; right panel: analysis of CBS mRNA level by qPCR, ***p < 0.0001. **c** SKOV3 Era-R cells were treated as in (**b**); GSH content and MDA were analysed, ***p < 0.0001. **d** SKOV3 Era-R cells were transfected with siCtrl or siNRF2 for 48 h, then treated with indicated doses of erastin for 24 h. Cell viability was examined, ***p < 0.0001. **e** Potential ARE sequence in the human CBS promotor region. **f** Left panel: Diagram illustrating cloning of wild-type and deletion mutant of CBS promoter fragments into pGL3-basic luciferase vector. Right panel: The indicated reporter vector, together with pCDNA3 empty vector or pCDNA3-Myc3-NRF2 and pCH110 (β-galactosidase-expressing plasmid) were cotransfected into SKOV3 Era-R cells. The activities of these reporter constructs were measured 24 h post transfection, **p < 0.001, ***p < 0.0001.

Accumulated evidences have positioned the antioxidant transcriptional factor NF-E2-related factor-2 (NRF2) to regulate ferroptosis through its target genes.^38^ To decipher how CBS was upregulated in erastin-resistant cells, we monitored NRF2, CBS and CSE abundance at different time points in response to erastin treatment. We observed a clear correlation with the induction of NRF2 and CBS as examined by immunoblotting (Fig. 5a). We noticed that erastin-resistant cells exhibited higher basal level of NRF2 than that of parental cells (Fig. 5 lanes 5 and 1), indicating the constitutive activation of NRF2 in erastin-resistant cells. To determine the role of NRF2 in the activation of CBS, we silenced NRF2 by siRNA and analysed CBS protein and mRNA expression. Silencing NRF2 dramatically declined CBS and prevented the activation of CBS upon erastin treatment (Fig. 5b). We further observed that the diminished GSH content and the increased MDA in NRF2-deficient cells (Fig. 5c), indicate the perturbation of cell redox homoeostasis and the increase in lipid peroxidation. Consequently, NRF2 depletion abrogated cells resistance to erastin (Fig. 5d). NRF2 regulates antioxidant gene expression via activation of antioxidant response elements (AREs).^39^ The previous study suggested that mouse CBS is regulated by NRF2, likely through an ARE in the upstream region of the gene.^40^ We wondered whether human CBS promoter contains ARE. In silico examination of the human CBS genomic locus revealed that it contains one putative ARE sequence located in the proximal promoter region, approximately from +67 to +97 bp, as illustrated in Fig. 5e. To determine whether this ARE is functional to mediate NRF2-dependent upregulation of CBS gene expression, we cloned CBS promoter fragment from −1000 to +200 bp and mutant fragment with deletion of +77 to +87 bp into a pGL3-basic luciferase reporter vector, which were designated as pGL3-phCBS and pGl3-phCBS-ΔARE, respectively. NRF2 overexpression in SKOV3 Era-R cells significantly increased the luciferase activity of reporter pGL3-phCBS plasmid, whereas depletion of the putative ARE in the promoter region of the CBS gene decreased the NRF2-induced luciferase reporter activity (Fig. 5f), indicating that the ARE is responsible for NRF2 regulation of CBS. Together, these results suggested that NRF2 was responsible for the upregulation of CBS in erastin-resistant cells.

## Discussion

**Fig. 6:**
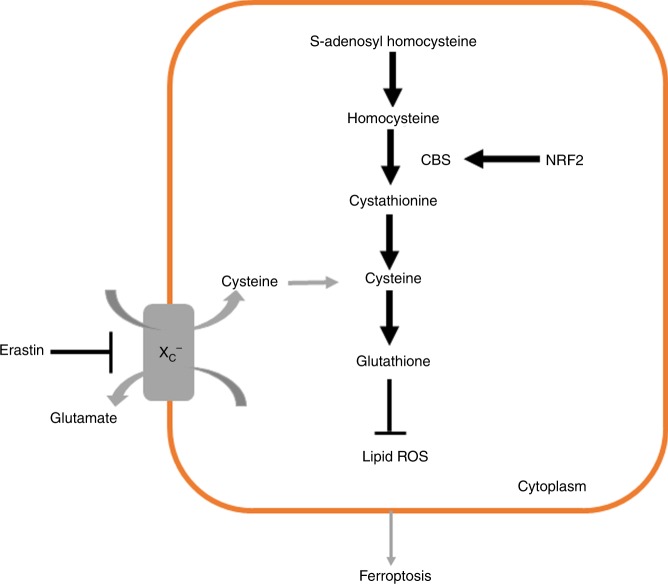
Schematic model of activation of NRF2/CBS axis regulating ferroptosis resistance.

Recent studies have shown the potential efficacy of ferroptosis inducer erastin in antitumour treatment^22,41–43^ and the synergism with chemotherapeutic agents in certain cancer cells.^32,44^ In an effort to investigate the effect of erastin in ferroptotic cell death in ovarian cancer cells, we found that erastin treatment could induce ferroptosis resistance. Upon erastin treatment, some cells exploited the transsulfuration pathway as a major source of cysteine, which counteracted the cysteine shortage due to system X_c_^−^ inhibition. We reported the regulatory role of the transsulfuration pathway in ferroptosis repertoire (Fig. 6).

The main treatment for ovarian cancer is debulking surgery followed by chemotherapy and/or radiation therapy. Despite an initially sensitive chemotherapy, most ovarian cancers relapse with chemoresistance, which is featured by genotypic alteration and metabolic changes.^45–47^ Thus, understanding the mechanisms during the progression to drug resistance may guide to develop effective therapeutic strategies to reduce ovarian cancer mortality. Ferroptosis, like apoptosis, is an exquisitely regulated cell death and is likely the adaptive process to remove malignant cells.^48–50^ We set out to search the potentially clinical use of ferroptosis inducers in cancer treatment. Notably, we observed that ferroptosis inducer erastin can also induce ferroptosis resistance in ovarian cancer cells. The finding is reminiscent of chemoresistance, which represents a major barrier for ovarian cancer therapy.

We further investigated the mechanism underlying ferroptosis resistance. In response to chronic erastin treatment, very few SKOV3 and OVCA429 cells developed the enhanced flux through the transsulfuration pathway and gradually adapted to cystine deprivation caused by system X_c_^−^ blockage. CBS catalyses the committing step in this pathway and is subject to multilevel regulation.^51^ Recent studies have shown the oncogenic role of CBS in colon and ovarian cancer models.^52–55^ We observed sustained upregulation of CBS in erastin-resistant cells, and further elucidated that upregulation of CBS was sufficient to render ferroptosis resistance. Knocking down CBS promoted cellular oxidative stress and lipid peroxidation, ultimately leading to ferroptosis. Overexpressing CBS enhanced the transsulfuration pathway and conferred ferroptosis resistance.

To examine how CBS was upregulated in erastin-resistant cells, we identified that NRF2 was constitutively activated and was positively correlated with CBS induction. We further identified a putative ARE in the human CBS promoter region and verified that ARE is responsible for NRF2-activated CBS promoter-driven luciferase activity. Furthermore, NRF2 inhibition caused CBS downregulation and sensitised cells to erastin result. We concluded that activation of NRF2/CBS accounts for ferroptosis resistance. We have not ruled out the possibility that other NRF2-targeted genes might be involved in ferroptosis resistance; additional studies will be required to investigate the role of NRF2 in the inhibition of ferroptosis.

## Data Availability

All presented data are available from the corresponding author upon reasonable request.

## References

[1] Bannai S, Christensen HN, Vadgama JV, Ellory JC, Englesberg E, Guidotti GG (1984). Amino acid transport systems. Nature.

[2] Bannai S, Kitamura E (1980). Transport interaction of L-cystine and L-glutamate in human diploid fibroblasts in culture. J. Biol. Chem..

[3] Banjac A, Perisic T, Sato H, Seiler A, Bannai S, Weiss N (2008). The cystine/cysteine cycle: a redox cycle regulating susceptibility versus resistance to cell death. Oncogene.

[4] Conrad M, Sato H (2012). The oxidative stress-inducible cystine/glutamate antiporter, system x (c) (-): cystine supplier and beyond. Amino Acids.

[5] Goji T, Takahara K, Negishi M, Katoh H (2017). Cystine uptake through the cystine/glutamate antiporter xCT triggers glioblastoma cell death under glucose deprivation. J Biol. Chem..

[6] Sato H, Tamba M, Ishii T, Bannai S (1999). Cloning and expression of a plasma membrane cystine/glutamate exchange transporter composed of two distinct proteins. J. Biol. Chem..

[7] Sato H, Tamba M, Kuriyama-Matsumura K, Okuno S, Bannai S (2000). Molecular cloning and expression of human xCT, the light chain of amino acid transport system xc. Antioxid. Redox. Signal..

[8] Dixon SJ, Lemberg KM, Lamprecht MR, Skouta R, Zaitsev EM, Gleason CE (2012). Ferroptosis: an iron-dependent form of nonapoptotic cell death. Cell.

[9] Xie Y, Hou W, Song X, Yu Y, Huang J, Sun X (2016). Ferroptosis: process and function. Cell Death Differ..

[10] Dixon SJ, Stockwell BR (2019). The Hallmarks of Ferroptosis. Ann. Rev. Cancer Biol..

[11] Friedmann Angeli JP, Krysko DV, Conrad M (2019). Ferroptosis at the crossroads of cancer-acquired drug resistance and immune evasion. Nat. Rev. Cancer.

[12] Proneth B, Conrad M (2019). Ferroptosis and necroinflammation, a yet poorly explored link. Cell Death Differ..

[13] McBean GJ (2012). The transsulfuration pathway: a source of cysteine for glutathione in astrocytes. Amino Acids.

[14] Sbodio JI, Snyder SH, Paul BD (2019). Regulators of the transsulfuration pathway. Br. J. Pharmacol..

[15] Stipanuk MH, Dominy JE, Lee JI, Coloso RM (2006). Mammalian cysteine metabolism: new insights into regulation of cysteine metabolism. J. Nutr..

[16] Kraus JP, Hasek J, Kozich V, Collard R, Venezia S, Janosikova B (2009). Cystathionine gamma-lyase: Clinical, metabolic, genetic, and structural studies. Mol. Genet. Metab..

[17] Bearden SE, Beard RS, Pfau JC (2010). Extracellular transsulfuration generates hydrogen sulfide from homocysteine and protects endothelium from redox stress. Am. J. Physiol. Heart. Circ. Physiol..

[18] Sugars KL, Rubinsztein DC (2003). Transcriptional abnormalities in Huntington disease. Trends Genet.

[19] Hensley K, Denton TT (2015). Alternative functions of the brain transsulfuration pathway represent an underappreciated aspect of brain redox biochemistry with significant potential for therapeutic engagement. Free Radic. Biol. Med..

[20] Zhang Yan, Tan Hui, Daniels Jacob D., Zandkarimi Fereshteh, Liu Hengrui, Brown Lewis M., Uchida Koji, O'Connor Owen A., Stockwell Brent R. (2019). Imidazole Ketone Erastin Induces Ferroptosis and Slows Tumor Growth in a Mouse Lymphoma Model. Cell Chemical Biology.

[21] Skouta R, Dixon SJ, Wang J, Dunn DE, Orman M, Shimada K (2014). Ferrostatins inhibit oxidative lipid damage and cell death in diverse disease models. J Am Chem Soc.

[22] Gout PW, Buckley AR, Simms CR, Bruchovsky N (2001). Sulfasalazine, a potent suppressor of lymphoma growth by inhibition of the x(c)- cystine transporter: a new action for an old drug. Leukemia.

[23] Tarangelo A, Magtanong L, Bieging-Rolett KT, Li Y, Ye J, Attardi LD (2018). p53 Suppresses metabolic stress-induced ferroptosis in cancer cells. Cell Rep.

[24] Dixon SJ, Patel DN, Welsch M, Skouta R, Lee ED, Hayano M (2014). Pharmacological inhibition of cystine-glutamate exchange induces endoplasmic reticulum stress and ferroptosis. Elife.

[25] Wang L, Cai H, Hu Y, Liu F, Huang S, Zhou Y (2018). A pharmacological probe identifies cystathionine beta-synthase as a new negative regulator for ferroptosis. Cell Death Dis..

[26] Fusco F, di Villa Bianca R, Mitidieri E, Cirino G, Sorrentino R, Mirone V (2012). Sildenafil effect on the human bladder involves the L-cysteine/hydrogen sulfide pathway: a novel mechanism of action of phosphodiesterase type 5 inhibitors. Eur. Urol..

[27] d’Emmanuele di Villa Bianca R, Mitidieri E, Di Minno MN, Kirkby NS, Warner TD, Di Minno G (2013). Hydrogen sulphide pathway contributes to the enhanced human platelet aggregation in hyperhomocysteinemia. Proc. Natl Acad. Sci. USA.

[28] Friedmann Angeli JP, Schneider M, Proneth B, Tyurina YY, Tyurin VA, Hammond VJ (2014). Inactivation of the ferroptosis regulator Gpx4 triggers acute renal failure in mice. Nat. Cell Biol..

[29] Slee EA, Zhu H, Chow SC, MacFarlane M, Nicholson DW, Cohen GM (1996). Benzyloxycarbonyl-Val-Ala-Asp (OMe) fluoromethylketone (Z-VAD.FMK) inhibits apoptosis by blocking the processing of CPP32. Biochem. J.

[30] Shimada K, Skouta R, Kaplan A, Yang WS, Hayano M, Dixon SJ (2016). Global survey of cell death mechanisms reveals metabolic regulation of ferroptosis. Nat. Chem. Biol..

[31] Marrocco I, Altieri F, Peluso I (2017). Measurement and clinical significance of biomarkers of oxidative stress in humans. Oxid. Med. Cell Longev..

[32] Sato M, Kusumi R, Hamashima S, Kobayashi S, Sasaki S, Komiyama Y (2018). The ferroptosis inducer erastin irreversibly inhibits system xc- and synergizes with cisplatin to increase cisplatin’s cytotoxicity in cancer cells. Sci. Rep..

[33] Singh S, Padovani D, Leslie RA, Chiku T, Banerjee R (2009). Relative contributions of cystathionine beta-synthase and gamma-cystathionase to H2S biogenesis via alternative trans-sulfuration reactions. J. Biol. Chem..

[34] Majtan T, Krijt J, Sokolova J, Krizkova M, Ralat MA, Kent J (2018). Biogenesis of hydrogen sulfide and thioethers by cystathionine beta-synthase. Antioxid. Redox Signal.

[35] Kimura Y, Goto Y, Kimura H (2010). Hydrogen sulfide increases glutathione production and suppresses oxidative stress in mitochondria. Antioxid. Redox Signal..

[36] Schreier SM, Muellner MK, Steinkellner H, Hermann M, Esterbauer H, Exner M (2010). Hydrogen sulfide scavenges the cytotoxic lipid oxidation product 4-HNE. Neurotox. Res..

[37] Beard RS, Bearden SE (2011). Vascular complications of cystathionine beta-synthase deficiency: future directions for homocysteine-to-hydrogen sulfide research. Am. J. Physiol. Heart Circ. Physiol..

[38] Abdalkader M, Lampinen R, Kanninen KM, Malm TM, Liddell JR (2018). Targeting Nrf2 to Suppress ferroptosis and mitochondrial dysfunction in neurodegeneration. Front. Neurosci..

[39] Kuosmanen SM, Viitala S, Laitinen T, Perakyla M, Polonen P, Kansanen E (2016). The effects of sequence variation on genome-wide NRF2 binding-new target genes and regulatory SNPs. Nucleic Acids Res..

[40] Hourihan, J. M., Kenna, J. G. & Hayes, J. D. The gasotransmitter hydrogen sulfide induces nrf2-target genes by inactivating the keap1 ubiquitin ligase substrate adaptor through formation of a disulfide bond between cys-226 and cys-613. *Antioxid. Redox Signal*. **19**, 465-481 (2013). 10.1089/ars.2012.4944 23145493

[41] Yu H, Guo P, Xie X, Wang Y, Chen G (2017). Ferroptosis, a new form of cell death, and its relationships with tumourous diseases. J. Cell. Mol. Med..

[42] Liu DS, Duong CP, Haupt S, Montgomery KG, House CM, Azar WJ (2017). Inhibiting the system xC(-)/glutathione axis selectively targets cancers with mutant-p53 accumulation. Nat. Commun..

[43] Yu Y, Xie Y, Cao L, Yang L, Yang M, Lotze MT (2015). The ferroptosis inducer erastin enhances sensitivity of acute myeloid leukemia cells to chemotherapeutic agents. Mol. Cell Oncol..

[44] Chen L, Li X, Liu L, Yu B, Xue Y, Liu Y (2015). Erastin sensitizes glioblastoma cells to temozolomide by restraining xCT and cystathionine-gamma-lyase function. Oncol. Rep..

[45] Schorge JO, McCann C, Del Carmen MG (2010). Surgical debulking of ovarian cancer: what difference does it make?. Rev. Obstet. Gynecol.

[46] Li SS, Ma J, Wong AST (2018). Chemoresistance in ovarian cancer: exploiting cancer stem cell metabolism. J. Gynecol. Oncol..

[47] Levine AJ, Puzio-Kuter AM (2010). The control of the metabolic switch in cancers by oncogenes and tumor suppressor genes. Science.

[48] Brasseur K, Gevry N, Asselin E (2017). Chemoresistance and targeted therapies in ovarian and endometrial cancers. Oncotarget.

[49] Lewerenz J, Ates G, Methner A, Conrad M, Maher P (2018). Oxytosis/Ferroptosis-(Re-) emerging roles for oxidative stress-dependent non-apoptotic cell death in diseases of the central nervous system. Front. Neurosci.

[50] Mou Y, Wang J, Wu J, He D, Zhang C, Duan C (2019). Ferroptosis, a new form of cell death: opportunities and challenges in cancer. J. Hematol. Oncol..

[51] Prudova A, Bauman Z, Braun A, Vitvitsky V, Lu SC, Banerjee R (2006). S-adenosylmethionine stabilizes cystathionine beta-synthase and modulates redox capacity. Proc. Natl Acad. Sci. USA.

[52] Phillips CM, Zatarain JR, Nicholls ME, Porter C, Widen SG, Thanki K (2017). Upregulation of cystathionine-beta-synthase in colonic epithelia reprograms metabolism and promotes carcinogenesis. Cancer Res..

[53] Jin S, Chen Z, Ding X, Zhao X, Jiang X, Tong Y (2016). Cystathionine-beta-synthase inhibition for colon cancer: Enhancement of the efficacy of aminooxyacetic acid via the prodrug approach. Mol Med.

[54] Bhattacharyya S, Saha S, Giri K, Lanza IR, Nair KS, Jennings NB (2013). Cystathionine beta-synthase (CBS) contributes to advanced ovarian cancer progression and drug resistance. PLoS One.

[55] Chakraborty PK, Xiong X, Mustafi SB, Saha S, Dhanasekaran D, Mandal NA (2015). Role of cystathionine beta synthase in lipid metabolism in ovarian cancer. Oncotarget.

